# 4129 Understanding Treatment Preferences for Hodgkin Lymphoma (HL) among Physicians, Patients and Caregivers

**DOI:** 10.1017/cts.2020.444

**Published:** 2020-07-29

**Authors:** Anita J Kumar, Rachel Murphy-Banks, John B Wong, Susan K Parsons

**Affiliations:** 1 Tufts University; 2 Tufts Medical Center

## Abstract

OBJECTIVES/GOALS: Although their 5-year survival >90%, young patients with HL face tradeoffs between near-term disease control and risk of treatment-related adverse effects decades later, so we seek to understand what patients and clinicians value in HL treatment decisions. METHODS/STUDY POPULATION: Leveraging our access to large cohorts of physicians, HL patients/survivors, and caregivers, we will use adaptive choice-based conjoint analysis (ACBC) to elicit treatment preferences when offered scenarios that incorporate tradeoffs, e.g., would a patient rather live 20 years with 10% risk of second malignancy or live 40 years with 30% of second malignancy. To reduce survey fatigue, prior choice responses limit subsequent scenarios. Through ACBC, we will identify variations in preferences and the importance of disease outcomes, treatment characteristics, and late effects for HL by respondent type. RESULTS/ANTICIPATED RESULTS: The goal is a final sample of 200 physicians and 200 patients/caregivers. We will collect demographics from physicians (age, type of physician, years practicing, type of practice, gender, and geography) and patients/caregivers (age at diagnosis, time since treatment, race, gender, smoker, education). We will ask questions about values of disease outcomes, late effects (second cancers, cardiac disease, chronic fatigue and neuropathy), and treatment characteristics (uncertainty of late effects, salvageability). Results will include utilities about participants views on disease-control and late effects. We anticipate participants to value disease control over late effects. DISCUSSION/SIGNIFICANCE OF IMPACT: Our study will elicit how physicians and patients/caregivers value treatment tradeoffs for HL. In an era of multiple treatment choices with varying short- and long-term benefits and harms, identifying values and preferences become critical for patient-centered treatment decisions.

